# Lactate exacerbates lung damage induced by nano-microplastics through the gut microbiota–HIF1a/PTBP1 pathway

**DOI:** 10.1038/s12276-024-01229-8

**Published:** 2024-05-29

**Authors:** Xin Chen

**Affiliations:** Department of Integrated Traditional Chinese and Western Medicine, Zigong First People’s Hospital, Zigong, China

**Keywords:** Drug development, Inflammation

Dear Editor,

We read and had great interest in the article written by Xuan et al.^1^. This study revealed that mice exhibited lung and intestinal mucosal damage, gut microbiota dysbiosis, and elevated lactate and N-cadherin levels after 28 days of exposure to nano-microplastics. In addition, lactate accumulation was strongly associated with an imbalance in lactic acid bacteria in the gut. Furthermore, no lactate accumulation was observed in germ-free mice, while depletion of the gut microbiota using a cocktail of antibiotics produced similar results. Mechanistically, elevated lactate level trigger activation of the HIF1a/PTBP1 pathway, which exacerbates nano-microplastic-induced lung damage through modulation of the epithelial–mesenchymal transition (EMT). However, we would like to discuss this topic further.

First, the sample size listed in the Methods section was significantly different from the number of samples displayed in the Results section. For example, there were 15 mice in each group in the antibiotic cocktail intervention experiment and 10 mice in each group in both the lactate intervention experiment and the PTBP1 knockout experiment. However, the number of samples included in the results was at most 5. We would like to know how the author calculated the sample size and why all samples were not tested and reported in the Results section. Second, the diversity within a given community (α diversity) is usually characterized using the total number of species (richness), the relative abundance of the species (evenness), or indices that combine these two dimensions. In addition, the partitioning of biological diversity among communities or along an environmental gradient (β diversity) is often characterized using the number of species shared between two communities^2^. Thus, the α and β diversity of the gut microbiome should be calculated and reported. Third, histological analysis revealed an increase in immune cell infiltration in the nano-microplastic group. Thus, the number of inflammatory cells and the expression of inflammatory cytokines such as IL-6 in bronchoalveolar lavage fluid should be evaluated between the different groups. Fourth, the authors detected different EMT biomarkers using different experimental methods in each experiment. For example, N-cadherin expression was detected by immunofluorescence analysis, as shown in Fig. 1e, f, but the relative expression levels of E-cadherin and N-cadherin, as shown in Figs. 3g, h and 4g, h; the relative expression levels of E-cadherin, N-cadherin and vimentin, as shown in Fig. 5d–f; and the relative expression levels of E-cadherin, as shown in Fig. 6e, were determined using real-time quantitative polymerase chain reaction (RT‒qPCR). However, the levels of N-cadherin protein expression in Fig. 6d were determined using Western blotting. Thus, why not use the same method to detect all three EMT biomarkers including E-cadherin, N-cadherin and vimentin in each experiment? Finally, all sequences of primers used in RT‒qPCR should be listed in the Methods section, but we found that the sequences of the primers of the endogenous reference was not included in the Methods section.
